# Establishment and validation of a two‐step screening scheme for improved performance of serological screening of nasopharyngeal carcinoma

**DOI:** 10.1002/cam4.1345

**Published:** 2018-02-25

**Authors:** Tingdong Li, Xiaoyi Guo, Mingfang Ji, Fugui Li, Han Wang, Weimin Cheng, Honglin Chen, Munhon Ng, Shengxiang Ge, Yong Yuan, Ningshao Xia

**Affiliations:** ^1^ State Key Laboratory of Molecular Vaccinology and Molecular Diagnostics National Institute of Diagnostics and Vaccine Development in Infectious Diseases School of Public Health and School of Life Science Xiamen University Xiamen Fujian China; ^2^ Cancer Research Institute of Zhongshan City Zhongshan Guangdong China; ^3^ State Key Laboratory for Emerging Infectious Diseases Department of Microbiology Li Ka Shing, Faculty of Medicine The University of Hong Kong Hong Kong SAR China

**Keywords:** Binary logistic regression, Epstein–Barr virus, nasopharyngeal cancer, positive predictive value, risk of NPC, serological screening

## Abstract

Nasopharyngeal carcinoma (NPC), which is closely associated with Epstein–Barr virus (EBV), is one of the most prevalent cancers in southeast China. Most NPC patients are diagnosed at late stage due to inconspicuous symptoms at the early stage, and the prognosis of these patients is poor. The early diagnosis rate of NPC could be significantly increased by serological screening, but the positive predictive value (PPV) is relatively low. A simple two‐step serological screening scheme was established to improve the PPV of the screening strategy and was validated by a prospective cohort. Serum antibodies specific for EBNA1, Zta, Thymidine Kinase (TK), EAD, EAR, and VCA were detected by enzyme‐linked immunosorbent assay. The combination of EBNA1/IgA and VCA/IgA was used in the first step of screening, and anti‐early antigens (EAs) were used in the second step of screening. EAD/IgA was the most prominent marker in the second step of screening, and other anti‐EAs were complementary to EAD/IgA. As validated by a prospective cohort including 4200 participants, using the combination of EAD/IgA and TK/IgA in the second step decreased the number of high‐risk participants from 128 to 27, and increased the PPV from 4.69% to 18.52%, with only one very early‐stage case missed. The two‐step screening scheme provides a standardized approach for NPC screening with an improved PPV and may be used in future field studies. With this two‐step serological screening method, more people benefit from the screening program without increasing the need for fiberoptic endoscopy.

## Background

Nasopharyngeal carcinoma (NPC), a common cancer in China and South‐East Asia, is closely associated with Epstein–Barr virus (EBV) 1. The 5‐year survival rate is greater than 90% for early‐stage NPC patients. However, due to inconspicuous symptoms at the early stage and the silent location of the anatomic site of the tumor, most NPC patients are diagnosed at late stages (III or IV), and the prognosis of these patients is poor 2. Therefore, screening for early‐stage NPC among the population is important.

Several anti‐EBV antibodies, especially immunoglobulin A (IgA), such as VCA/IgA, EA‐D/IgA, and EBNA1/IgA, were found to be higher in NPC patients compared to those of healthy carriers and patients with other head and neck diseases 3, 4, 5, 6, 7, 8, 9, 10. Since the 1970s, immunoglobulin A (IgA) antibodies to EBV capsid antigen (VCA/IgA) and EBV early antigen (EA/IgA) have been used for NPC screening in southern China. In early years, cell‐based serological techniques (immunofluorescence assay, IFA) were used for NPC screening. However, IFA is arduous, time‐consuming, and poorly standardized, making it less applicable for screening in large populations 11, 12, 13. Later, enzyme‐linked immunosorbent assay (ELISA) kits for anti‐EBV antibodies were developed and show better performance in NPC detection 14, 15, 16, 17, 18, 19, 20, 21. In addition, it has been found that combination of different antibody markers could improve the sensitivity and specificity of the serological screening, in which the most widely used combination was EBNA 1/IgA and VCA/IgA 22, 23, 24.

In 2008, a randomized large population screening program was launched in Zhongshan, China, which includes 28,688 participants and the combined EBNA1/IgA and VCA/IgA ELISA was used for serological screening. In this program, 862 participants were predicted to have a high risk of NPC, and 856 of these high‐risk participants were followed up and further evaluated by fiberoptic endoscopy and biopsy. In this subsequent screening program, 38 participants in the high‐risk group developed NPC, while only three participants in the low‐risk group developed NPC, and 65.9% (27/41) of the subjects were diagnosed at early stage (Stage I and II according to the TNM classification system). However, although the specificity was 97.12%, which is very high compared to that of other cancer markers, the positive predictive value was only 4.41% (38/862) even in the high prevalence areas 25. A total of 95.59% of the high‐risk participants required further diagnostic examinations. Fiberoptic endoscopy and biopsy examinations are invasive and expensive, and the results are subjective and highly dependent on the experience of the clinicians performing and interpreting the tests.

In 2009, Paramita et al. 17 found that 19 of 22 false positives in EBNA1/IgA and VCA/IgA results were normal in the EA/IgA assessment, suggesting that combing EA/IgA with EBNA1/IgA and VCA/IgA may increase the specificity of NPC screening. In this study, the antibodies specific for the four recombinant EA proteins (Zta, TK, EA‐D‐p54, and EA‐R‐p38) were detected in 46 screened NPC patients and 263 serologically defined high‐risk subjects using the combination of EBNA1/IgA and VCA/IgA from the previous screening program 25. A two‐step screening scheme was established based on differences in the serum antibodies specific for TK, EAD, and EAR. As validated by a prospective cohort, 128 of 4200 participants were predicted to have a high risk of NPC in the first step of screening using the combination of EBNA1/IgA and VCA/IgA, including six who were confirmed to have NPC by fiberoptic endoscopy and biopsy. With this two‐step serological screening method, more people could benefit from the screening program without increasing the need for fiberoptic endoscopy.

## Materials and Methods

### Study population

For the establishment of the two‐step screening scheme, 46 serum samples of screened NPC patients and 263 serum samples of serologically defined high‐risk participants using the combination of EBNA1/IgA and VCA/IgA from previous cohort studies started in 2008 25 and the subsequent screening programs. All patients were newly diagnosed with histologically confirmed nonkeratinizing NPC, and blood was collected at the first visit of the screening. A total of 57 NPC cases were identified in the screening program in Zhongshan, and 46 NPC cases with more than 200 *μ*L of serum remaining were selected for this study. 401 non‐NPC high‐risk cases were identified in the 2008–2009 screening program in Zhongshan, and 263 cases with more than 200 *μ*L of serum remaining were selected for this study. The disease status was assessed according to the tumor bulk (T), regional lymph node involvement (N), and metastasis (M), as determined by the staging system of 2008 26. Of the 46 screened NPC patients, 29 were diagnosed at an early stage (stage I&II) (63.04%). All serum samples were stored at −20°C until use.

To validate the performance of the two‐step screening scheme, a new prospective cohort study was initiated in 2015 in Zhongshan, China. A total of 4200 participants were enrolled, including 128 who were predicted to have a high risk of NPC as determined using the combination of EBNA1/IgA (Zhongshan Bio‐Tech company, Zhongshan, China) and VCA/IgA (EUROIMMUN AG, Lübeck, Germany) 25. All of the participants with a high risk of NPC were invited for fiberoptic endoscopy examination, and the serum antibodies specific for the early antigens of EBV were further assessed. All of the participants were informed about the study, and the study was approved by the medical ethics committee of the Cancer Research Institute of Zhongshan City, China.

### Serological antibody detection and risk prediction

For the first step of screening, serum samples were evaluated by the combination of EBNA1/IgA and VCA/IgA (EUROIMMUN AG, Lübeck, Germany) according to the product manual. The logistic regression PROB was calculated by the formula Logit *P* = 4.797*EBNA1/IgA +2.203*VCA/IgA‐3.934, and the participants with a PROB equal to or greater than 0.98 were defined as high risk 25.

For the second step of screening, the serum IgA and IgG antibodies of the high‐risk participants defined in the first step were detected using the recombinant early antigens (EA) Zta, TK, EAD, and EAR. The gene encoding the full length of Zta, TK, EAD‐p54, and EAR‐p30 was amplified from B‐95. The gene encoding Zta and EAD‐p54 was cloned into pGEX‐2T (GE Healthcare, Buckinghamshire, England), and the gene encoding TK was cloned into pCold^™^ TF (Takara, Tokyo, Japan), while EA‐R was cloned into pET‐30a‐c(+) (Novagen, Darmstadt, Germany). The expression plasmids carrying the EBV genes were transformed to *Escherichia coli* ER2566 (NEB, Ipswich, MA) for expression. After addition of IPTG, the bacteria were cultured at 25°C for another 5 h for expression. The recombinant proteins were purified from the lysis supernatant to homogeneity by Glutathione affinity chromatography (Zta and EAD‐p54) or Nickel NTA agarose affinity chromatography (TK and EAR‐p30) as described in the manufacturer's protocol.

The purified recombinant protein was diluted to 0.25 *μ*g/mL in 50 mmol/L carbonate buffer, pH 9.6, and was used at 100 *μ*L per well for antigen immobilization in a 96‐well polystyrene microplate at 37°C for 2 h, and blocked with 20% NBS in 20 mmol/L PBS at 37°C for another 2 h to minimize nonspecific binding. The serum samples were diluted 1:10 with sample dilution buffer (1% BSA, 10% newborn bovine serum and 0.1% Triton X in PBS), and 100 *μ*L of 1:10 diluted serum samples was added to the microplates precoated with recombinant Zta, TK, EAD, or EAR and incubated at 37°C for 30 min. After washing, the HRP‐conjugated secondary antibody specific for human IgG (KPL, Gaithersburg, Maryland, diluted at 1: 5000) or IgA (KPL, Gaithersburg, Maryland, diluted at 1: 20,000) was added to the microplates for the detection of IgG or IgA, respectively. After 15 min of color development and termination using a TMB substrate kit (WANTAI, Beijing, China), the optical density (OD) at a wavelength of 450 nm with a reference filter of 620 nm was determined using a Universal Microplate Reader (BioTek, Winooski, VT).

For quantification of the antibody titers, a pooled NPC serum sample was used as reference. The antibody titer of the reference was determined by twofold serial dilution from 1:20, and the endpoint dilution with a OD_450/620_ above the cutoff value was considered as the titer of the reference serum (Table S1). The cutoff value was determined as the mean OD_450/620_ of the EBV antibody negative controls plus 2 standard deviation (SD), and if the calculated cutoff was lower than 0.1, 0.1 was considered as the cutoff. For each plate, a serially 1.5‐fold diluted reference serum sample was detected in duplicate, and the standard curves were plotted using the log_2_‐transformed OD_450/620_ versus the antibody titer of each dilution by a linear regression, and the linear range was defined when the broadest range was included with *R* ^2^ above 0.99 (Table S1).

A serum sample with an OD_450/620_ below 0.1 was considered negative, and the titer was defined as 1:5. For a serum sample with a OD_450/620_ within the linear range of the standard curve, the antibody titer was calculated from the corresponding standard curve multiplied by 10; while for a serum sample with a OD_450/620_ above the standard curve, it will be 10‐fold serially diluted and tested again, and the antibody titer was calculated from the corresponding standard curve multiplied by the corresponding dilution fold.

To predict the risk of NPC, binary logistic regression and receiver operating characteristic (ROC) analysis were used as described in the statistical analysis.

### Fiberoptic endoscopy and biopsy

The participants in the prospective cohort with a high risk of NPC were invited for fiberoptic endoscopy examination by the local otorhinolaryngologists at the People's Hospital in Zhongshan within 6 months of the baseline test. Nasopharyngeal biopsies were retrieved if suspicious lesions were observed during the endoscopy. Histopathologically diagnosed patients were immediately given recommendations for treatment.

### Statistical analysis

The statistical analysis was performed using SPSS 18.0 (SPSS Inc., Chicago, IL).

The Mann–Whitney test was used to compare the antibody titers between the NPC group and the non‐NPC group. For the comparison of the differences in the sensitivity, specificity, and PPV, Pearson chi‐square test was used, while for the comparison of the sensitivity in the prospective cohort study, Fisher exact test was used. Statistical significance was set at *P* < 0.05. A binary logistic regression model was used to identify an optimal biomarker panel to discriminate NPC subjects from non‐NPC subjects with a high risk of NPC. MedCalc Statistical Software version 16.2.1 (MedCalc Software bvba, Ostend, Belgium; https://www.medcalc.org; 2016) was used to plot ROC curves. The area under the curve (AUC) was used to summarize the performance of the anti‐EBV IgA and IgG antibodies for differentiating NPC and non‐NPC populations with a high risk of NPC. In addition, the sensitivity and specificity were also determined by the results of ROC analysis. The PPV was calculated as the percentage of the participants confirmed to have NPC in the group of participants with positive serological results.

## Results

### Detection of serum anti‐EA antibody levels in screening NPC and non‐NPC high‐risk populations

**Figure 1:**
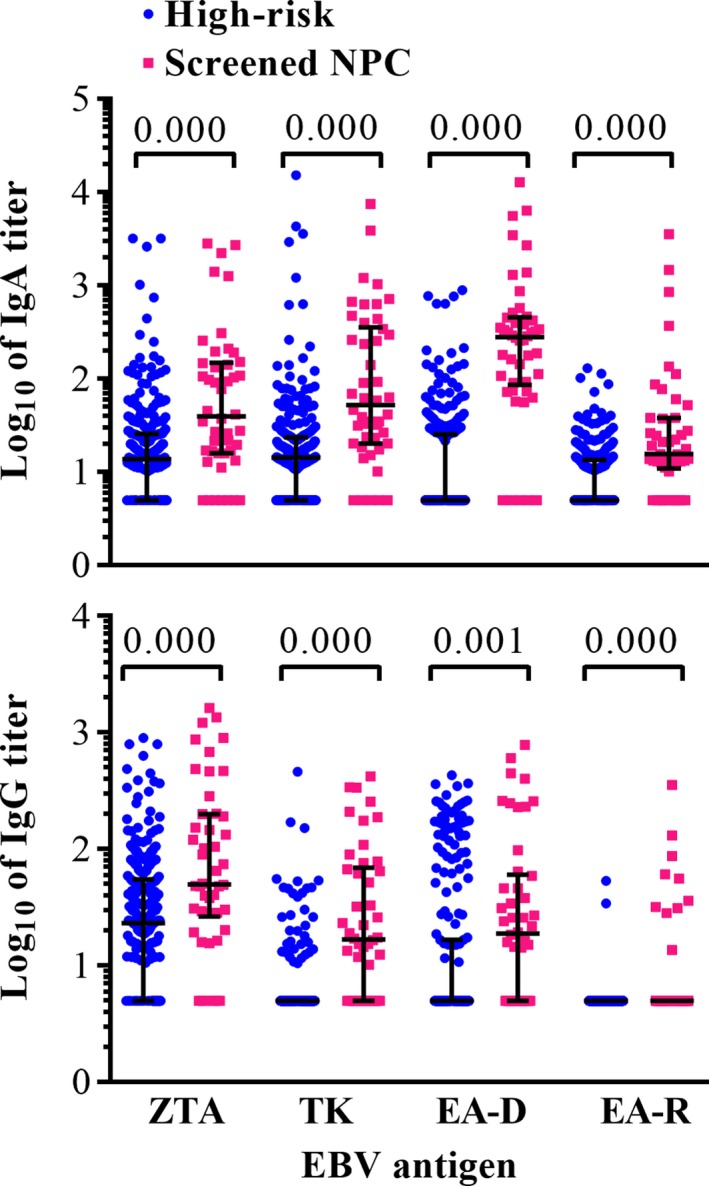
The anti‐EAs in NPC patients and non‐NPC high‐risk participants with elevated EBNA1/IgA and VCA/IgA. The medians and interquartile ranges of the antibody titers are shown in black line. The cases with OD_450/620_ value lower than the cutoff were considered as negative, and the antibody titer was defined as half the lowest dilution (1:5).

In the present cross‐sectional study, the serum anti‐EA IgA and IgG levels of 46 screened NPC patients and 263 non‐NPC participants with elevated EBNA1/IgA and VCA/IgA in the screening program and confirmed as negative by endoscopy were determined. The results showed that the serum antibody levels in the NPC subjects were higher than those in the non‐NPC subjects (Fig. 1 and Table 1). The positive rates of anti‐EAD IgA and IgG, anti‐EAR IgA and IgG, and anti‐TK IgG were lower than 50% in the non‐NPC participants with elevated EBNA1/IgA and VCA/IgA (Table 1). These results suggested that the combination of these antibodies with the previously determined combination of EBNA1/IgA and VCA/IgA may improve the specificity of the serological screening.

**Table 1:** The performance of different anti‐EBV antibodies in distinguishing NPC (*n* = 46) from non‐NPC participants with an elevated risk as determined by the combination of EBNA1/IgA and VCA/IgA (*n* = 263)

Antibody	AUC (95% CI)	Highest sensitivity^a^	Cutoff^b^	Specificity	Complementary antibody	AUC (95% CI)
Zta/IgA	0.723 (0.636–0.809)	***84.78% (39/46)***	1:11.08	38.78% (102/263)	EAD/IgA	0.896 (0.856–0.929)
TK/IgA	0.790 (0.710–0.870)	***89.13% (41/46)***	1:5	31.84% (84/263)	EAD/IgA	***0.925 (0.889–0.952)***
EAD/IgA	***0.902 (0.841–0.963)***	***89.13%*** (41/46)	1:20	***73.38% (193/263)***	TK/IgG	***0.928 (0.890–0.953)***
EAR/IgA	0.753 (0.672–0.835)	78.26% (36/46)	1:5	60.84% (160/263)	EAD/IgA	***0.930 (0.893–0.956)***
Zta/IgG	0.705 (0.620–0.790)	***86.96% (40/46)***	1:15.48	40.30% (106/263)	EAD/IgA	0.892 (0.851–0.925)
TK/IgG	0.786 (0.701–0.871)	67.39% (31/46)	1:5	***86.69% (228/263)***	EAD/IgA	***0.928 (0.890–0.953)***
EAD/IgG	0.656 (0.569–0.742)	63.04% (29/46)	1:11.59	73.00% (192/263)	EAD/IgA	0.873 (0.830–0.909)
EAR/IgG	0.605 (0.506–0.704)	21.74% (10/46)	1:5	***99.24% (261/263)***	EAD/IgA	0.894 (0.853–0.927)

a Sensitivity is defined as the positive rate of each assay with a cutoff of 1:5.

b The cutoff was defined as the highest antibody titer not higher than 1:20 without decreasing the sensitivity.

Receiver operating characteristic analysis was used to analyze the performance of different anti‐EBV antibodies in distinguishing NPC and non‐NPC high‐risk populations, and EAD/IgA was found to be the most prominent marker, with an AUC of 0.903 (0.843–0.964), followed by TK/IgA, TK/IgG, and EAR/IgA (Table 1). The performance of EAD/IgA was significantly higher than that of TK/IgA (*P* = 0.005). The highest sensitivity was 89.13% when these markers were applied separately (Table 1).

### The combination of different EA‐specific antibodies in distinguishing NPC and non‐NPC high‐risk populations

As shown in Table S2, among the 71 EAD/IgA‐positive but non‐NPC(FP) cases, 13, 24, and 55 subjects were TK/IgA‐, EAR/IgA‐, and TK/IgG‐negative, respectively, while in the 5 EAD/IgA‐negative NPC cases, four, four, and two subjects were TK/IgA‐, EAR/IgA‐, and TK/IgG‐positive. Similar complementary results were observed for other antibody markers (Table S2), suggesting that the combination of these antibodies may improve the sensitivity and specificity of the serological screening program. As analyzed via binary logistic regression, TK/IgG was found to be the most effective complementary marker for EAD/IgA, while EAD/IgA was the most effective complementary marker for TK/IgA, EAR/IgA, and TK/IgG (Table 1). The formulas of logit *P* were as follows: 2.433*EAD/IgA+0.537*TK/IgA‐6.354, 2.457*EAD/IgA+0.794* EAR/IgA ‐6.495, and 2.374*EAD/IgA+1.805*TK/IgG‐7.256 for the three combinations, respectively.

### The performance of different combinations in distinguishing NPC and non‐NPC high‐risk populations

**Figure 2:**
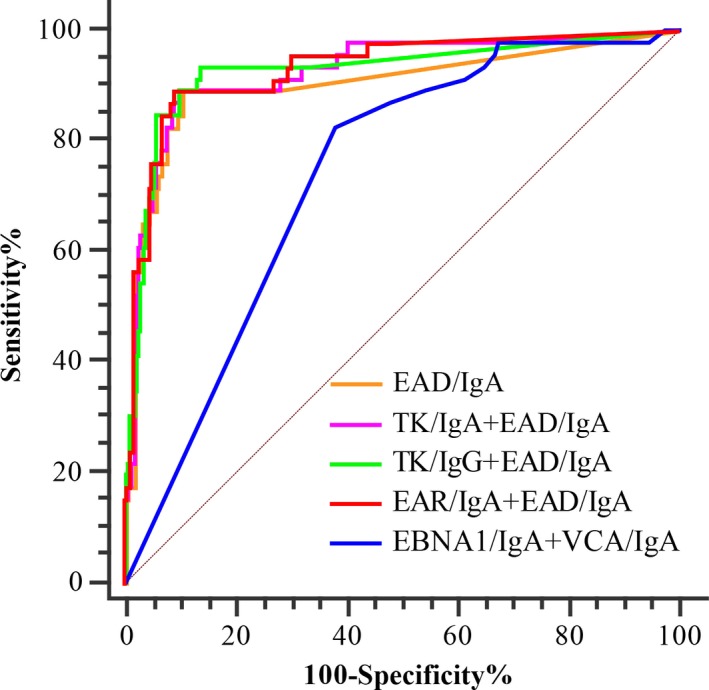
ROC analysis of different combinations in distinguishing NPC from non‐NPC high‐risk populations. The control group was defined as the individuals with a PRE value above 0.98 in the first step of screening using the combination of EBNA1/IgA+VCA/IgA.

The titers of EAD/IgA and the PROB were used for the ROC analysis. The AUC values were 0.902, 0.925, 0.928, and 0.930 for EAD/IgA and the three combinations of TK/IgA+EAD/IgA, EAD/IgA+TK/IgG, and EAR/IgA+EAD/IgA, respectively (Fig. 2 and Table 1). The AUCs of the three combinations were slightly higher than that of EAD/IgA alone (*P* > 0.10), but they were significantly higher than those of other antibody markers alone (*P* < 0.0001) (Table 1). No significant differences were observed in the performance of the three combinations (Table 1), while the AUC of EAD/IgA and the three combinations were significantly higher that of EBNA1/IgA+VCA/IgA used in the first‐step screening (*P* < 0.001, Fig. 2).

By combining these antibody markers, the highest sensitivity could be increased to 97.83%, 97.83%, and 93.48% for the combinations of TK/IgA+EAD/IgA, EAD/IgA+EAR/IgA, and EAD/IgA+TK/IgG, respectively, and no significant differences were observed compared to the sensitivity of EAD/IgA (*P* = 0.091, 0.091, and 0.459, respectively). At the highest sensitivity, the highest specificity was 48.67% (cutoff = 0.018), 51.71% (cutoff = 0.018), and 85.93% (cutoff = 0.135) for the three combinations, respectively. The specificity of EAD/IgA+TK/IgG was significantly higher than those of the other two combinations and EAD/IgA (73.38%) (*P* < 0.001). For a specificity of 85.93%, the sensitivity of both combinations will decrease to 89.13%, which is slightly lower than that of EAD/IgA+TK/IgG (*χ*² = 0.173, *P* = 0.7114). With a cutoff of 1:39.3, the specificity of EAD/IgA could also be increased to 85.93% with the sensitivity of 89.13% (Table 2). Therefore, the combination of EAD/IgA with TK/IgG, the most efficient complementary marker, but not TK/IgA or EAR/IgA could improve the performance of the second step of screening. With the sensitivity of 92.68% for the first‐step screening, the overall sensitivity could be 82.74% and 86.78% for the two‐step screening when EAD/IgA or the combination of EAD/IgA+TK/IgG was used for the second step screening, respectively. In addition, the NPC cases were relatively low than the performance of EAD/IgA and the combination of EAD/IgA+TK/IgG should be further evaluated.

**Table 2:** The performance of different antibody combinations in distinguishing NPC (*n* = 46) from non‐NPC participants with an elevated risk as determined by the combination of EBNA1/IgA and VCA/IgA (*n* = 263)

Antibody	AUC (95% CI)	Highest sensitivity			Specificity = 85.93%	
		Cutoff	Sensitivity (95% CI)	Specificity (95% CI)	Cutoff	Sensitivity (95% CI)
EAD/IgA	0.902 (0.841–0.963)	1:20	89.13% (76.4%–96.4%)	73.38% (67.6%–78.6%)^a^	1:39.3	89.13% (76.4%–96.4%)
EAD/IgA+TK/IgA	0.925 (0.889–0.952)	0.018	97.83% (88.5%–99.9%)	48.67% (42.5%–54.9%)^a^^,^ b	0.158	89.13% (76.4%–96.4%)
EAD/IgA+EAR/IgA	0.930 (0.893–0.956)	0.018	97.83% (88.5%–99.9%)	51.71% (45.5%–57.9%) a ^**,**^ b	0.161	89.13% (76.4%–96.4%)
EAD/IgA+TK/IgG	0.928 (0.890–0.953)	0.135	93.48% (82.1%–98.6%)	85.93% (81.1%–89.9%)	0.135	93.48% (82.1%–98.6%)

a The specificity was significantly lower than that of the combination of EAD/IgA+TK/IgG.

b The specificity was significantly lower than that of EAD/IgA.

### Validation of the two‐step screening scheme by a prospective cohort

**Figure 3:**
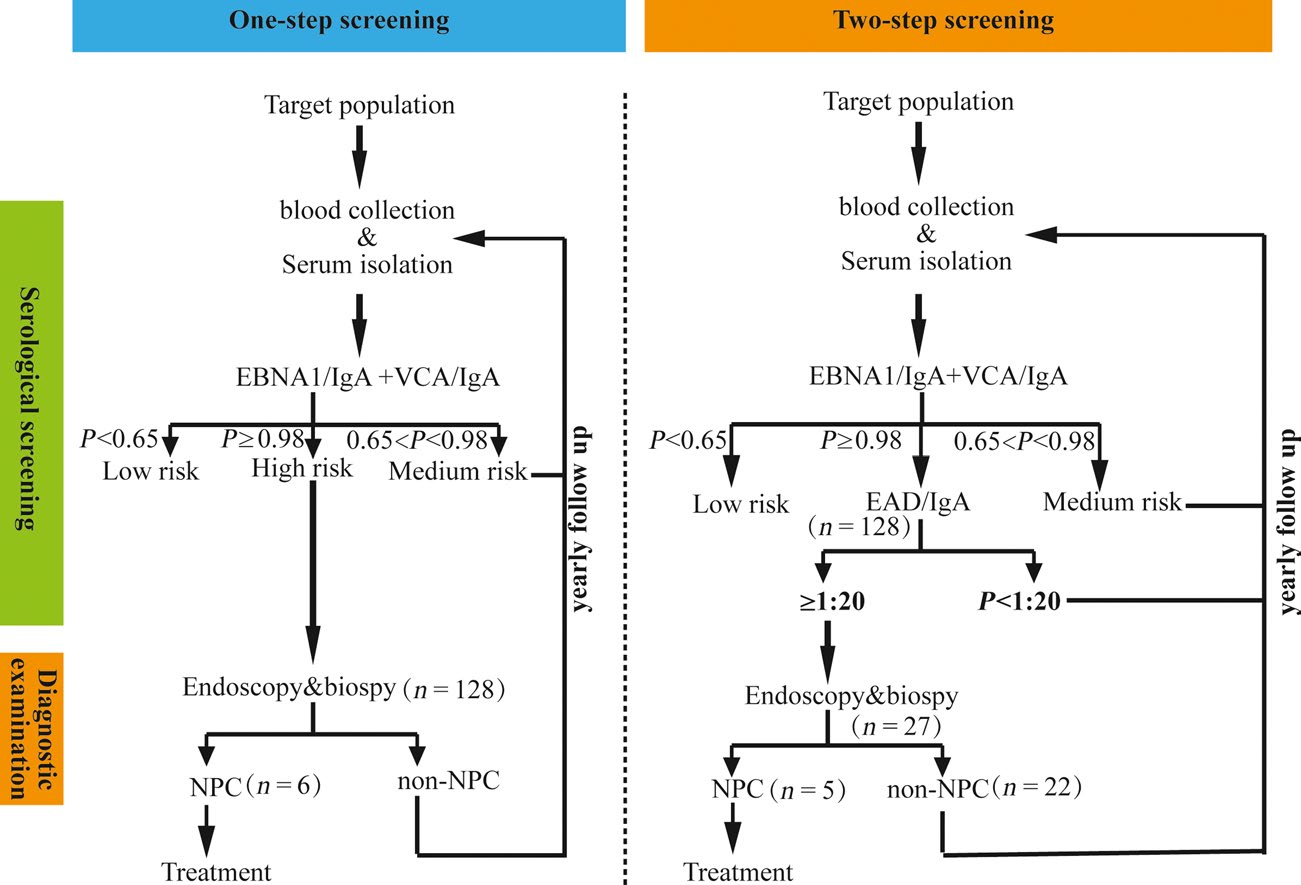
The schemes of the one‐step and two‐step screening methods for NPC. The data shown in the figure were derived from the prospective cohort study.

To validate the performance of the two‐step screening scheme in the screening program, a new prospective cohort study was initiated in Zhongshan, China. The 128 participants with a high risk of NPC were simultaneously invited for fiberoptic endoscopy examination and the second step of serological screening, and a total of six participants were confirmed to have NPC by fiberoptic endoscopy and biopsy. When only EAD/IgA was considered at a cutoff of 1:20, similar to that in the initial study (specificity = 73.38%), there was a 78.91% (101/128) reduction in the high risk. Five of the NPC cases confirmed by diagnostic examination were in this group, and the subsequent sensitivity was 83.33% (5/6) and the PPV could be increased from 4.69% (6/128) in the first step of screening to 18.52% (5/27) (Table 3). The NPC participants with a negative EAD/IgA result were diagnosed at very early stage, with weakly positive TK/IgG result; however, with a PROB of 0.025, the missed NPC case could not be screened out by this combination with the predetermined cutoff. As shown in Table 3, the high‐risk cases could be decreased to 24, which is slightly less than that when EAD/IgA was used alone. However, another NPC case could be missed using this combination as it was TK/IgG‐negative (Table 3). Considering these results, EAD/IgA would be the best choice for the second step of screening. The overall sensitivity was 83.3%, which does not significantly decrease compared to the first‐step screening alone (Fisher's exact test, *P* = 1.00). While the specificity increased from 97.09% in the first step of screening to 99.48% (*χ*² = 70.66, *P* < 0.001), and the PPV of the two‐step screening was also significantly higher, increased from 4.69% to 18.52% (*χ*² = 4.54, *P* = 0.033). The two‐step screening scheme was summarized in Figure 3.

**Table 3:** The performance of the two‐step screening method in the prospective cohort

Antibody	Cutoff	High risk		Reduction in high risk^a^ (%)	Reduction in NPC^b^ (%)	PPV^c^	NPV^d^
		NPC	Non‐NPC				
EBNA1/IgA+VCA/IgA	0.98	6	122	0 (100%)	0 (100%)	4.69% (6/128)	100% (4072/4072)
EAD/IgA	1:20	5	22	101 (78.91%)	1 (16.7%)	18.52% (5/27)^e^	99.98% (4172/4173)
EAD/IgA	1:39.3	4	15	109 (85.16%)	2 (33.3%)	21.05% (4/19)^e^	99.95% (4179/4181)
EAD/IgA+TK/IgG	0.135	4	20	104 (81.25%)	2 (33.3%)	16.67% (4/24)^e^	99.95% (4172/4173)

a The reduction in high risk was calculated as the number of high‐risk cases defined in the first step of screening‐the number of high‐risk cases in the second step of screening, while the percentage of the reduction was calculated as (1‐the number of high‐risk cases defined in the second step/128) *100%.

b The reduction in NPC was calculated as the number of NPC cases after diagnostic examination‐the number of NPC cases identified in the second step of screening, while the percentage of the reduction was calculated as (1‐the number of NPC cases in the second step/6) *100%.

c PPV was calculated as (the number of NPC cases after diagnostic examination/the number of high risk after the second step of screening) *100%.

d NPV was calculated as (the number of non‐NPC participants not predicted to be high risk/the number of participants not predicted to be high risk) *100%.

e P<0.05 as compared to EBNA1/IgA+VCA/IgA.

## Discussion

Early‐stage diagnosis and treatment are critical to improve the survival rates of cancer patients, including those with NPC 26, a cancer with a high incidence in South‐East Asia, such as southern China and Singapore 27. It has been reported that the 5‐year survival rate is significantly higher when NPC is diagnosed at an early stage 28; however, most NPC cases are diagnosed at a late stage due to the inconspicuous nature of early‐stage symptoms 2. Anti‐EBV antibodies are higher in NPC patients, and it has been found that antibodies may be elevated 1 year prior to the development of NPC 29, allowing the early diagnosis of NPC. In a large cohort study, the early‐stage diagnosis rate could be increased to 68.3% using a combination of two serological antibody tests, EBNA1/IgA and VCA/IgA 25. However, the PPV is relatively low (4.41%) 25 and limits the screening population size because of the dependence on experienced otorhinolaryngologists for confirmation. If the specificity of the screening could be further improved, more people could benefit from the screening program.

In August 2017, Chan et al. 30 found that with a persistent positive result of EBV DNA in plasma (4‐week interval), the sensitivity and specificity of EBV DNA in the prediction of NPC were 97.1% and 98.6%, respectively, with a PPV of approximately 11% (34/309). In principle, there are no significant differences between DNA screening and serological screening as the persistent replication of the EBV could also stimulate antibody responses, resulting in elevated antibodies. Although the sensitivity of serological screening may be slightly lower, however, this method could be more cost‐effective for the following reasons. First, the cost of the serological test is significantly lower than that of DNA test: the serological test costs approximately 5 US$, while the DNA test costs approximately 30 US$/test. Second, the serological test was more automated than the DNA test, and the test throughput was higher than that of the DNA test. Third, the serological test saved time compared to the DNA test. In addition, the environmental requirements are more stringent for DNA test because it is usually carried out in a separate space as the DNA test can be easily contaminated.

In the present study, we reported an improved PPV of NPC screening by a two‐step serological screening scheme, with the combination of EBNA1/IgA and VCA/IgA in the first step and anti‐EA antibodies in the second step. Although one very early‐stage NPC case was missed, the PPV could be increased up to 18.52% and only 27 of the 128 high‐risk participants required further diagnostic examination (Table 3). As we know, fiberoptic endoscopy examination is time‐consuming and the accuracy of the results is highly dependent on the experience of the otorhinolaryngologists, limiting the number of subjects who can be screened. With the second step of serological screening, the number of screened individuals could be increased fourfold without increasing the number of experienced otorhinolaryngologist, indicating that if 100,000 people could be screened previously, then 400,000 people could be screened with the two‐step screening scheme. Although some very early cases of NPC could be missed in the second step of screening (one of six in this study), the progression of NPC is slow and so it may not be too late when subjects are screened during the follow‐up.

The concept of “two‐step screening” was proposed by Paramita et al. 17 in 1999, and the complementary effects of IgA (EBNA1 + VCA) and EA/IgA have been observed in Indonesian participants. However, samples with a clear background (NPC or not) were used in that study, and the anti‐EAs of both the false‐negative samples and the false‐positive samples in the first step of detection were reevaluated. In the screening program, only the high‐risk samples in the first step of screening could be assessed in the second step as the NPC background was unknown. Despite the differences in the two‐step screening, the results of Paramita et al. and our own results showed that the antibodies specific for EA were complementary to the antibodies specific for EBNA1 and VCA. Moreover, in contrast to Paramita et al., recombinant EAs but not native EAs were used in this assay, and the antibodies to different EAs were complementary as analyzed by logistic regression (Table 1), while EAD/IgA alone was sufficient for the second stage of screening. The scheme of the two‐step screening is summarized in Figure 3.

Although the performance of the screening could also be improved by the combination of the anti‐EAs with EBNA1/IgA and VCA/IgA, this is arduous and significantly the more expensive. By two‐step screening, only the samples of high‐risk participants (approximately 3% of the entire screening population) in the first step should be detected in the second step, improving the performance and mitigating increases in detection costs and workload. On the other hand, increasing the cutoff of the PROB using the combination of EBNA1/IgA and VCA/IgA could also increase the specificity of the screening; however, to achieve the same specificity as the two‐step screening scheme, the sensitivity would decrease from 92.68% to 70.37% (Fig. 2). Therefore, the performance of the two‐step screening scheme should be better than that of the one‐step screening scheme.

In conclusion, the specificity of serological screening of NPC could be improved by a two‐step screening scheme without significantly decreasing the sensitivity, which would benefit more people. However, there were some sensitivity differences in the establishment and validation of the scheme, which may be due to the relatively small number of NPC cases in the validation cohort (6/128). Therefore, the sensitivity and cost‐effectiveness of EAD/IgA in the second step should be further analyzed using a larger population. In addition, the antibody titers were determined using an ELISA assay, which could be arduous in large‐scale screening. To address this issue, an automated chemiluminescence assay could be developed. Moreover, the molecular diversity of anti‐EBV antibodies in Indonesian, Chinese and European subjects has been reported in previous studies 31, and the prevalence of NPC varies across regions. Therefore, the performance of the two‐step screening scheme in different areas should be further validated by field studies.

## Conflict of Interest

The authors declare no potential conflict of interests.

## Supporting information


**Table S1**. The performance of the anti‐EA assays.
**Table S2.** Complementary of different anti‐EA antibodies in distinguishing NPC from non‐NPC high‐risk population.
**Figure S1**. SDS‐PAGE and western blotting characterization of the purified recombinant EBV antigens.
