# Supplementary material for: Establishment and validation of a two‐step screening scheme for improved performance of serological screening of nasopharyngeal carcinoma
Source: Cancer Med. 2018 Feb 25;7(4):1458–67. doi: 10.1002/cam4.1345 (PMC5911604; doi:10.1002/cam4.1345)
Supplement: Supplementary file 1 — Table S1. The performance of the anti‐EA assays. Table S2. Complementary of different anti‐EA antibodies in distinguishing NPC from non‐NPC high‐risk population. Figure S1. SDS‐PAGE and western blotting characterization of the purified recombinant EBV antigens. [file CAM4-7-1458-s001.docx]

Table S1. The performance of the anti-EA assays

| Antibody | Linear range ^a^ | Titer of reference | CV% at low titer (GMT) | CV% at medium titer (GMT) | CV% at high titer (GMT) |
| --- | --- | --- | --- | --- | --- |
| Zta/IgA | 1:10-1:200 | 1:320 | 3.68 (10.42) | 4.44 (53.44) | 4.88 (116.93) |
| TK/IgA | 1:10-1:200 | 1:160 | 5.98 (10.13) | 3.94 (53.66) | 8.02 (118.48) |
| EAD/IgA | 1:10-1:200 | 1:640 | 3.79 (10.52) | 7.48 (54.85) | 7.34 (152.33) |
| EAR/IgA | 1:10-1:200 | 1:160 | 4.64 (10.54) | 5.79 (55.69) | 8.58 (126.75) |
| Zta/IgG | 1:10-1:200 | 1:160 | 2.50 (24.61) | 2.01 (52.36) | 8.43 (181.53) |
| TK/IgG | 1:10-1:200 | 1:160 | 3.45 (22.77) | 2.79 (52.21) | 3.20 (119.12) |
| EAD/IgG | 1:10-1:200 | 1:80 | 2.97 (23.14) | 2.22 (52.38) | 2.69 (120.40) |
| EAR/IgG | 1:10-1:200 | 1:80 | 2.85 (25.18) | 2.41 (47.87) | 1.06 (139.50) |

^a^: For each plate, a serially 1.5-fold diluted reference serum sample was detected in duplicate, and the standard curves were plotted using the log2 transformed OD_450/620_ vs the antibody titer of each dilution by a linear regression, and the linear range was defined when the broadest range was included with R2 above 0.99, for each of the assay, the reference sample was diluted to a titer of 1:200 for the first dilution.

^b^: The anti-EA antibody titer of a pooled NPC serum sample was determined by 2-fold serial dilution, and the endpoint dilution with an OD_450/620_ above the cut-off was defined as its antibody titer.

Table S2 Complementary of different anti-EA antibodies in distinguishing NPC from non-NPC high-risk population

| Status ^a^ | | Number | TK/IgA | |  | EAD/IgA | |  | EAR/IgA | |  | TK/IgG | |
| --- | --- | --- | --- | --- | --- | --- | --- | --- | --- | --- | --- | --- | --- |
|  |  |  | + | - |  | + | - |  | + | - |  | + | - |
| TK/IgA | FP | 179 | 179 | 0 |  | 58 | **121** |  | 90 | **89** |  | 31 | **148** |
|  | FN | 5 | 0 | 5 |  | **4** | 1 |  | 1 | 4 |  | 2 | 3 |
| EAD/IgA | FP | 71 | 58 | 13 |  | 71 | 0 |  | 47 | **24** |  | 16 | **55** |
|  | FN | 5 | **4** | 1 |  | 0 | 5 |  | **4** | 1 |  | 2 | 3 |
| EAR/IgA | FP | 103 | 90 | 13 |  | 47 | **56** |  | 103 | 0 |  | 20 | **83** |
|  | FN | 10 | 6 | 4 |  | **9** | 1 |  | 0 | 10 |  | 4 | 6 |
| TK/IgG | FP | 35 | 31 | 4 |  | 16 | **19** |  | 20 | 15 |  | 35 | 0 |
|  | FN | 15 | **12** | 3 |  | **12** | 3 |  | 9 | 6 |  | 0 | 15 |

^a^: FP represents false positive, eg. Positive as detected by indicated antibodies of the non-NPC participants defined as high-risk population by the serological screening using EBNA1/IgA and VCA/IgA; FN represents false negative, eg. Negative as detected by indicated antibodies of the NPC patients.


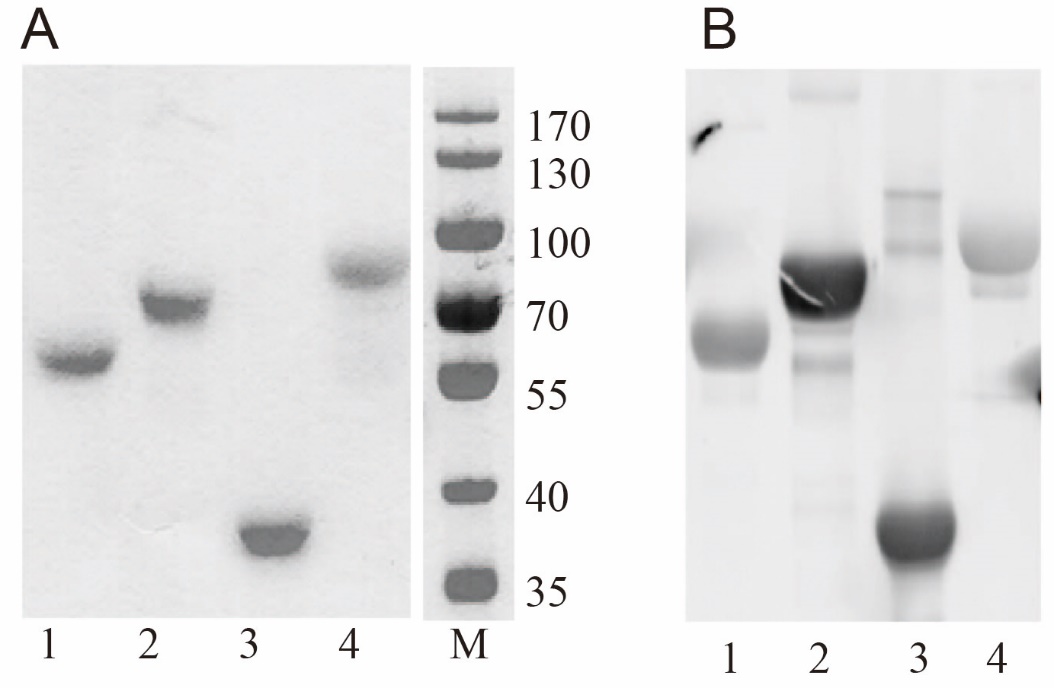


Fig. S1. SDS-PAGE and western blotting characterization of the purified recombinant EBV antigens. A. The purified proteins were analyzed by 12% SDS-PAGE; B. The purified proteins were analyzed by western blot using pooled serum samples from NPC patients. lane 1 to 4: Zta, EA-D, EA-R, TK, Lane M: protein molecular marker.
